# Enlargements of the Spleen—II

**Published:** 1908-08-01

**Authors:** 


### Medicine.

### ENLARGEMENTS OF THE SPLEEN—II.

### THE PHYSICAL SIGNS (*continued*).

III. *Percussion.*—(*a*) On percussion the enlarged spleen yields a dull note unless the force of percussion is considerable; and the dull note obtained over the abdominal tumour is continuous with an increased area of dulness in the lower part of the left thorax behind and at the side, extending upwards as high as the seventh rib in the mid-axiliary line, and the sixth rib in the left nipple line, or even higher.

(*b*) On percussing the left loin a resonant note should be elicited, showing that the descending colon has not been displaced.

(*c*) No intestines can be made out to be lying in front of the tumour by palpation or by percussion.

IV. *Auscultation.*—(*a*) On auscultation, if perisplenitis is associated with the enlargement, a loud friction sound may be heard. This is not uncommon in cases of splenic infarction.

(*b*) In some cases, especially if the enlargement is associated with venous engorgement, or with dilatation of the veins, a continuous humming or blowing bruit may be heard over the mass.

It will be noted that palpation is of more value than the other three groups of physical signs put together in determining the existence of a splenic enlargement. Percussion comes next in usefulness, inspection is sometimes of service, whilst auscultation is the least use of all.

### Tumours that may be Mistaken for Enlarged Spleen.

Enlargement of the spleen has to be distinguished from other tumours which arise in the left hypochondriac region, such as (1) kidney tumours of all kinds; (2) suprarenal tumours; (3) perinephric abscess; (4) malignant growth of the splenic flexure of the colon; (5) pancreatic tumours, especially cysts, but also new growths; (6) ovarian cysts; (7) fæces in the bowel; (8) malignant growth of the stomach; (9) tuberculous peritonitis.

*Splenic* versus *Renal Tumours.*—The greatest of all the above difficulties is the distinction between enlargements of the spleen and of the left kidney. The points to which particular attention should be directed in making the diagnosis are the following:—

(*a*) Both renal and splenic enlargements may cause a local prominence or bulging of the left side of the abdomen. In the case of a splenic enlargement the bulging is more forward and in the anterior and inward direction, whereas in a kidney enlargement the loin is more likely to be bulged. It is important to examine the patient in the sitting posture if possible, so as to compare the two loins as they are to be seen when looked at from above.

(*b*) No distinct edge or notch can be seen or felt in the case of a renal enlargement, a most important point, the significance of which cannot be over-estimated. A renal enlargement presents a general rounded conformation, with smooth rounded protuberances from the main mass in some cases of new growth or of cystic disease.

(*c*) A kidney tumour may move downwards when the patient takes a deep breath, but the extent of the movement is much less than is that of an enlarged spleen. This difference is due to the fact that the spleen is in much closer relationship to the undersurface of the diaphragm than is the kidney.

(*d*) A renal tumour is more deeply situated in the abdomen, and does not approximate closely to the anterior abdominal wall unless the enlargement is very great indeed.

(*e*) Posteriorly a kidney tumour fills out the loin, which consequently feels firm and resistant.

(*f*) A renal tumour generally slopes away towards the ribs, so that the hand can be placed between it and the costal margin.

(*g*) A renal tumour hardly ever extends across the middle line.

(*h*) Occasionally the descending colon can be seen or felt lying over the anterior surface of a renal tumour.

(*i*) A kidney enlargement usually yields a resonant note on percussion anteriorly, on account of the position of the colon, which has been pushed forward by the enlargement of the organ, and so lies in front of it.

(*j*) Posteriorly, on percussing the left loin, a dull note is elicited, as the colon has been pushed forward, and its position taken by the enlarged kidney.

(*k*) A vertical band of resonance due to the colon may sometimes be detected very clearly down the front of the otherwise dull renal tumour; this is never the case with a splenic tumour.

(*l*) Neither bruit nor rub are at all likely to be heard over a renal tumour.

Although there are so many points of difference between a renal and a splenic tumour, it is not always a simple matter to distinguish between them. In the majority of cases careful attention to the physical examination and a critical investigation of the history of the case and of the above-mentioned points will suffice. There are, however, instances in which it is impossible from physical examination of the abdomen alone to say whether the tumour is an enlargement of tne kidney or of the spleen. A careful examination of the blood on the one hand for anæmia or leucocytosis, or of the urine on the other for pus, blood, albumen, casts, crystals, bacilli, or fragments of new growth may be required.

It must be remembered, however, that the detection of even a marked leucocytosis may be an insufficient ground for diagnosis of enlarged leuchæmic spleen. A differential leucocyte count is required as well, or else, as in the following instance, a case of pyonephrosis and perinephric abscess may be misdiagnosed as one of leuchæmia:—

*Perinephric Abscess Mistaken for Enlarged Spleen.*—The patient was a lady, aged 56, who complained of pain and swelling in the left side of her abdomen, just below the ribs. She was married, and had one child, aged 15. She stated that she had suffered from pain in her back for more than 20 years. About the middle of March 1899 she became very ill and prostrate. She consulted a doctor, who examined her, and told her there was some enlargement of the left kidney. When seen as an out-patient at a hospital on April 17, 1899, there was a large tumour occupying the left hypochondriac, left lumbar, and left half of the umbilical regions. The mass was tender, and it presented a fairly well-defined hard rounded edge, which extended from the tip of the ninth left costal cartilage downwards, and inwards to a point about an inch below the umbilicus. On bimanual palpation the tumour could be felt to extend far back into the loin, but also close up under the ribs in front. On percussion a dull note was found over its anterior surface, continuous with what appeared to be an increased area of splenic dulness. A fresh specimen of blood was examined, and an enormous increase in the number of white blood corpuscles was found; there appeared to be about one white to every eight or ten red cells. The character of the tumour and the leucocytosis led to a diagnosis of leuchæmia. The patient was admitted to the ward, and the diagnosis remained at leuchæmia until a fuller examination of the blood demonstrated that the leucocytosis, though considerable, was diminishing, and that in the differential leucocyte count there were no myelocytes, and a very high proportion of polymorphonuclear cells. A little later redness and œdema of the loins developed, the tumour suddenly became resonant, a large quantity of pus was passed per anum, and the patient died on May 22, after losing quantities of blood through the bowel.

At the post-mortem examination a small calculus was found in the pelvis of the left kidney, together with a large pyonephrosis and perinephric abscess. The latter had opened into the colon at the splenic flexure, and the abscess cavity was full of blood: this had come from an erosion at the lower end of the spleen which was forming the upper boundary of the abscess.

